# Influences of carrier sex, body size, and time on the symbiotic interaction between *Nicrophorus vespilloides* and the *Uroobovella nova* mite species complex

**DOI:** 10.1038/s41598-025-04685-y

**Published:** 2025-06-05

**Authors:** Daria Bajerlein, Piotr Zduniak, Aleksandra Wyszyńska, Edward Baraniak, Marek Przewoźny, Tomasz Grzegorczyk, Arkadiusz Urbański

**Affiliations:** 1 Department of Animal Taxonomy and Ecology, Faculty of Biology, Adam Mickiewicz University, Poznań, Poland, Uniwersytetu Poznańskiego 6, Poznań, 61-614 Poland; 2 Department of Avian Biology and Ecology, Faculty of Biology, Adam Mickiewicz University, Poznań, Poland, Uniwersytetu Poznańskiego 6, Poznań, 61-614 Poland; 3 Department of Systematic Zoology, Faculty of Biology, Adam Mickiewicz University, Poznań, Poland, Uniwersytetu Poznańskiego 6, Poznań, 61-614 Poland; 4 Department of Animal Physiology and Developmental Biology, Faculty of Biology, Adam Mickiewicz University, Poznań, Poland, Uniwersytetu Poznańskiego 6, Poznań, 61-614 Poland

**Keywords:** Burying beetles, Dispersal, Mites, Phoresy, Symbiosis, Ecology, Zoology

## Abstract

Phoretic dispersal is critical in low-mobile invertebrates because it enables feeding, breeding, and gene flow. Phoresy may have serious evolutionary consequences for species in highly specific interactions. Mites within the *Uroobovella nova* species complex have a narrow range of carriers limited to burying beetles. *Nicrophorus vespilloides*, a model organism used in behavioural studies, is a common carrier of *U*. *nova*, but this interaction remains underexplored. This study investigated how carrier sex, body size, season, and year affect the relationship between *U*. *nova* and *N. vespilloides*. We tested the hypotheses that mite infestation is sex-biased because of differences in parental care between females and males and that larger individuals carry more mites. Mite prevalence was affected only by season. A slightly higher mite load was found in females than in males, and mites showed a significant but weak preference for beetle body size. Considerable temporal differences in mite load were found. Deutonymphs were highly specific when selecting attachment sites, irrespective of the carrier sex, and appeared on some body parts when the preferred sites had already been infested. The low specificity of *U*. *nova* towards *N. vespilloides* individuals and the high selectivity of attachment sites seem to increase the probability of colonising beetle brood chambers.

**Supplementary Information:**

The online version contains supplementary material available at 10.1038/s41598-025-04685-y.

## Introduction

Burying beetles (*Nicrophorus* spp.) are known for using small carrion and providing extensive biparental care to their offspring. For this reason, they have become model systems in evolutionary studies of sexual conflict or parent‒offspring competition and are among the best-studied beetles [e.g.^1–5^]. Another well-known feature of *Nicrophorus* species is that they frequently carry mites. Their symbiotic relationships with representatives of the genus *Poecilochirus* Canestrini & Canestrini, 1882 (Parasitidae) are currently among the best-studied examples of phoretic dispersal [e.g.^6–11^]. Burying beetles are also frequently used as a means of transport by mites within the *Uroobovella nova* species complex^12–14^. Surprisingly, this phoretic relationship has been neglected compared with studies of *Poecilochirus* mites and burying beetles.

The *Uroobovella nova* species complex is classified within Uropodina mites, which disperse phoretically by coexisting insects, particularly beetles [for a review, see^15^]. Phoresy is critical for Uropodina because it enables the colonisation of new habitats, further development, feeding, and breeding. Phoretic Uropodina, similarly to other phoretic animals, do not incur parasitic costs to their carriers as parasites do. In most species, dispersal occurs at the deutonymphal stage, which has evolved morphological and behavioural adaptations for phoresy^16^. Under unfavourable conditions, deutonymphs actively seek their carriers and attach to them via a pedicel, i.e., a temporarily secreted structure that looks like an elongated stalk with two termini^16–18^.

*Uroobovella nova* (Oudemans, 1902) was originally described as *Uroseius novus*, but Knee et al.^14^. revealed that this species represents at least five morphologically similar species with relatively restricted host ranges. The authors suggested that the species *Uroobovella* sp. 3. is the closest to *U. nova* originally described by Oudemans^19^ because it covers carrier species (*Nicrophorus vespilloides* Herbst, 1783 and *Nicrophorus vespillo* (Linnaeus, 1758)) and geographic distribution (European countries)^14^.

Unlike most species of Uropodina, which have a wide range of carriers, including species within one, two or even several unrelated families, mites within the *U*. *nova* species complex disperse exclusively on burying beetles (*Nicrophorus* spp.)^14,15^. To date, *U*. *nova* mites have been the subject of mostly genetic and morphological analyses verifying their taxonomic status^12,14^. Moreover, morphological analyses of pedicels secreted by deutonymphs have been conducted^18^. The only ecological study that provided insight into the nature of the phoretic relationships between *U*. *nova* and burying beetles was conducted by Schwarz et al.^13^. The authors studied the carrier specificity of *U*. *nova*, the level of deutonymph infestation, and the deutonymph’s preferred attachment sites. The temporal variation in phoresy in *U*. *nova* mites and the carrier-dependent factors affecting deutonymph infestation have never been investigated.

This paper presents novel data on the phoretic associations between *U*. *nova* mites and *N*. *vespilloides.* More specifically, we tested whether the prevalence and intensity of deutonymph infestations are affected by the season, year, sex, and body size of *N. vespilloides*. We also compared the deutonymph load according to infested beetle body parts and examined whether deutonymph attachment sites are sex-biased. We also verified whether the deutonymphs are evenly distributed between the left and right carrier body sides and tested how the number of carried deutonymphs affects the number of infested beetle body parts.

The following hypotheses were tested: (1) Deutonymph infestation is sex-biased because of the different behaviours of females and males of *N. vespilloides*. Current research shows that although both parents provide extensive care in the brood chamber, males usually abandon offspring two or three days earlier than females do^1,20^. Furthermore, females spend more time on food provision and parental care than males do^21^. Therefore, the exposure of mites to different sexes can be expected. (2) Larger beetle individuals have higher mite loads. Previous studies revealed that phoretic mites are more successful on larger carriers. Moreover, burying beetle behaviour is characterised by high plasticity and is strongly associated with beetle body size^22^. (3) The number of carried deutonymphs is affected by the season and year. Recent research by Issar et al.^23^ revealed that the reproductive success and number of larvae produced by *N. vespilloides* may change during the year. Therefore, temporal differences in the deutonymph loads collected from beetles can be expected. (4) The number of infested beetle body parts increases with increasing deutonymph load. Some body parts of *N. vespilloides* are selected by deutonymphs only because the preferred parts have already been infested.

## Results

### Deutonymph prevalence

**Fig. 1:**
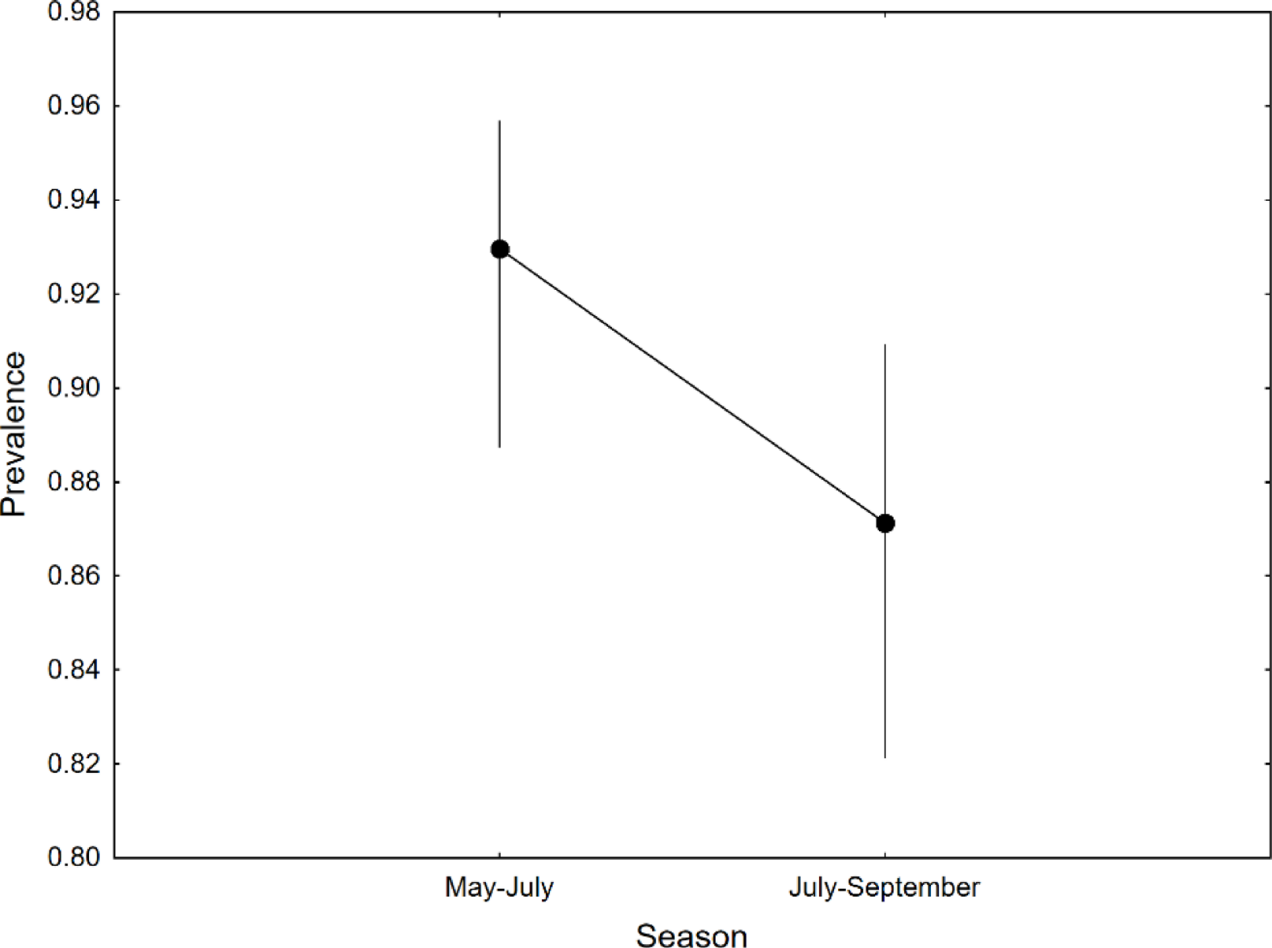
Prevalence of deutonymphs within the *Uroobovella nova* species complex carried by *Nicrophorus vespilloides* with relation to the season; mean values are presented with 95% confidence limits.

Phoretic deutonymphs were found in 89.3% (95% CL: 86.21–91.95; *n* = 427) of *N*. *vespilloides* individuals. The infestation status of the beetles varied between SEASONS, but it did not depend on their SEX, SIZE or YEAR (Table 1; Fig. 1, Supplementary Table S1).

**Table 1:** Summary of generalized linear model (GLM) analysis of the factors influencing the prevalence of deutonymphs from the *Uroobovella nova* species complex phoretic on *Nicrophorus vespilloides*.

Factor	Wald χ2	df	*p*
INTERCEPT	0.01	1	0.943
SEX	0.57	1	0.450
BODY SIZE	1.90	1	0.168
SEASON	4.22	1	0.040
YEAR	2.36	1	0.124
SEASON*YEAR	1.28	1	0.257
SEX*SEASON*YEAR	2.57	1	0.109

### Intensity of deutonymph infestation

**Fig. 2:**
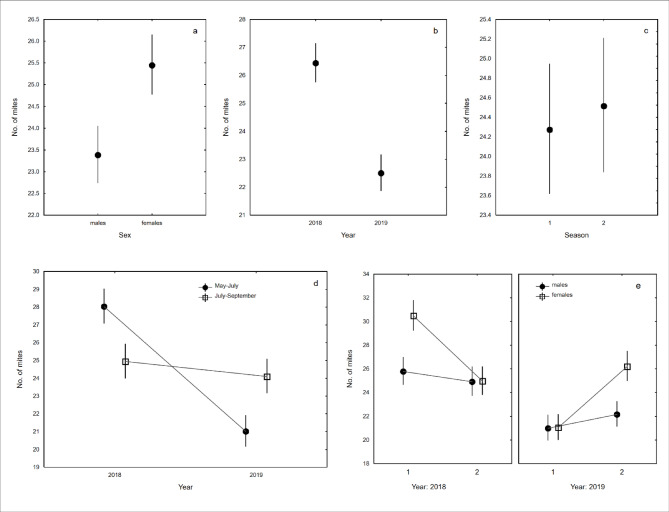
Intensity of infestation of deutonymphs within the *Uroobovella nova* species complex carried by *Nicrophorus vespilloides* in relation to sex (**a**), year (**b**), season (**c**; 1–May–July, 2–July–September), interaction between season and year (**d**), and the interaction between sex, season, and year (**e**; 1–May–July, 2–July–September); mean values are presented with 95% confidence limits.

The intensity of deutonymph infestation was 24.5 (CL: 22.7–26.4, range: 1–148, *n* = 427). Overall, females carried, on average, 8.8% more mites than males did (Table 2; Fig. 2a). Furthermore, the number of mites was positively but weakly related to the SIZE of the beetles (*β* = 0.05, CL: 0.01–0.09; Table 2). Moreover, the intensity of mite infestations differed between the YEARS, with more mites collected in 2018, but not between the studied SEASONS (Table 2; Fig. 2b,c). The interaction effect between SEASON and YEAR was also significant (Table 2; Fig. 2d). The highest intensity of mite infestations was found in beetles collected in May–July 2018, followed by those collected in July–September 2018 and July–September 2019, with the lowest intensity occurring in May–July 2019 (Table 2; Fig. 2d). In addition, the interaction effect of SEX, SEASON, and YEAR was also statistically significant (Table 2; Fig. 2e). The highest intensity of deutonymph infestation was found in females in May–July of 2018. Mite loads were similar in males collected during both seasons in 2018 and in females collected in July–September of 2018 and 2019. The lowest intensity of deutonymph infestation was observed in both seasons of 2018 for males and females collected in May–July 2018 (Fig. 2e).

**Table 2:** Summary of the generalized linear model (GLM) analysis used to explore the factors influencing the intensity of infestations of deutonymphs from the *Uroobovella nova* species complex phoretic on *Nicrophorus vespilloides*.

Factor	Wald χ^2^	df	*p*
INTERCEPT	699.05	1	< 0.001
SEX	18.33	1	< 0.001
BODY SIZE	4.02	1	0.045
SEASON	0.25	1	0.620
YEAR	65.70	1	< 0.001
SEASON*YEAR	40.70	1	< 0.001
SEX*SEASON*YEAR	17.31	1	< 0.001

### Deutonymph distribution on the beetle carrier

**Fig. 3:**
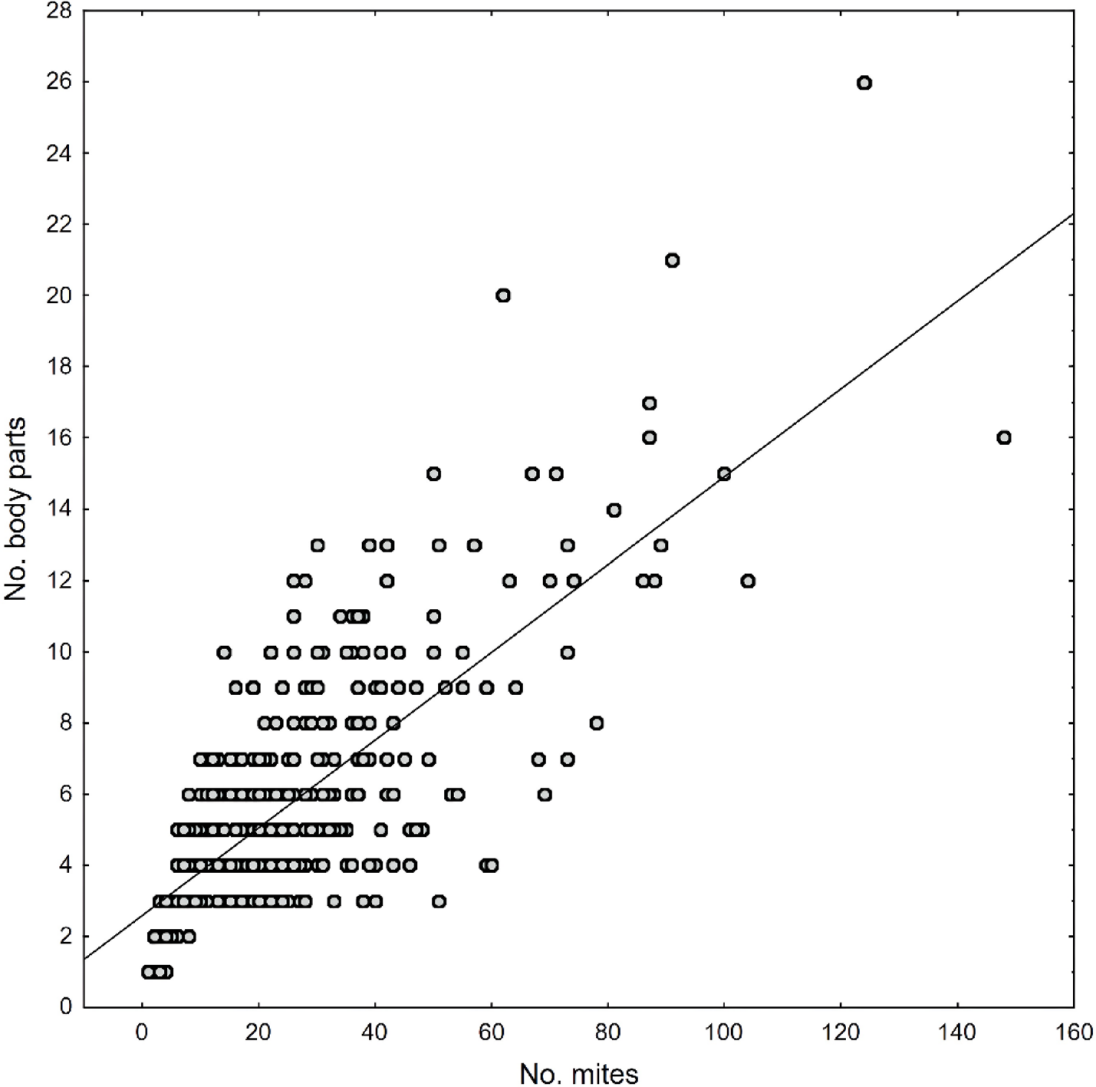
Pearson’s correlation between the number of phoretic deutonymphs within the *Uroobovella nova* species complex recorded on *Nicrophorus vespilloides* and the number of beetle body parts to which the mites were attached; y = 0.12x + 2.58, *n* = 427.

**Fig. 4:**
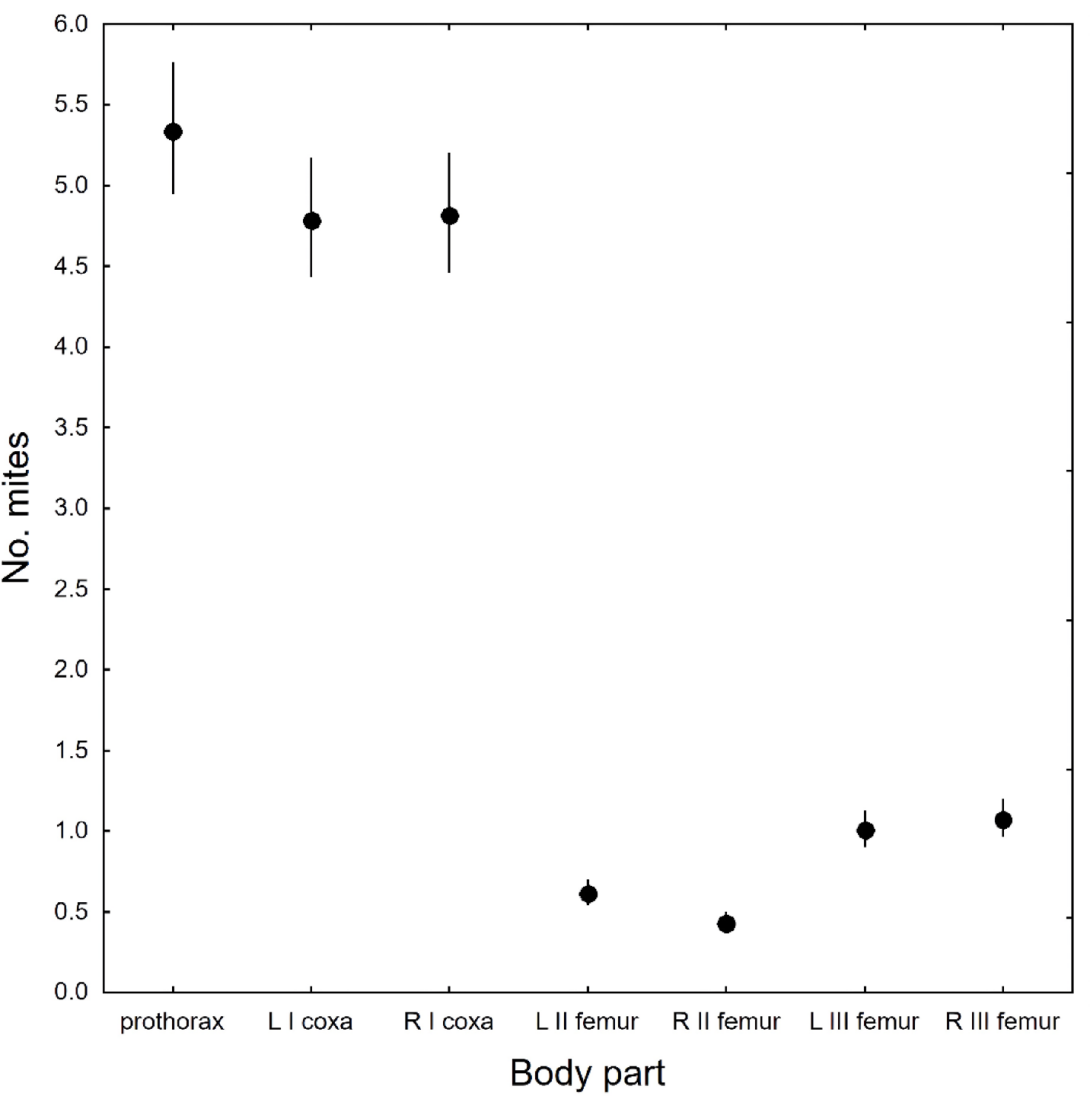
Mean numbers of mites of the *Uroobovella nova* species complex attached to the most heavily infested *Nicrophorus vespilloides* body parts considering the left (L) and right (R) body sides; the means are presented with 95% confidence limits; the overall mean value for all beetle body parts was 3.1, CL: 3.0–3.3, *n* = 2989. I – forelegs, II – midlegs, III – hindlegs, coxa – the ventral side of the coxa, femur – the dorsal side of the femur; mean values are presented with 95% confidence limits.

**Fig. 5:**
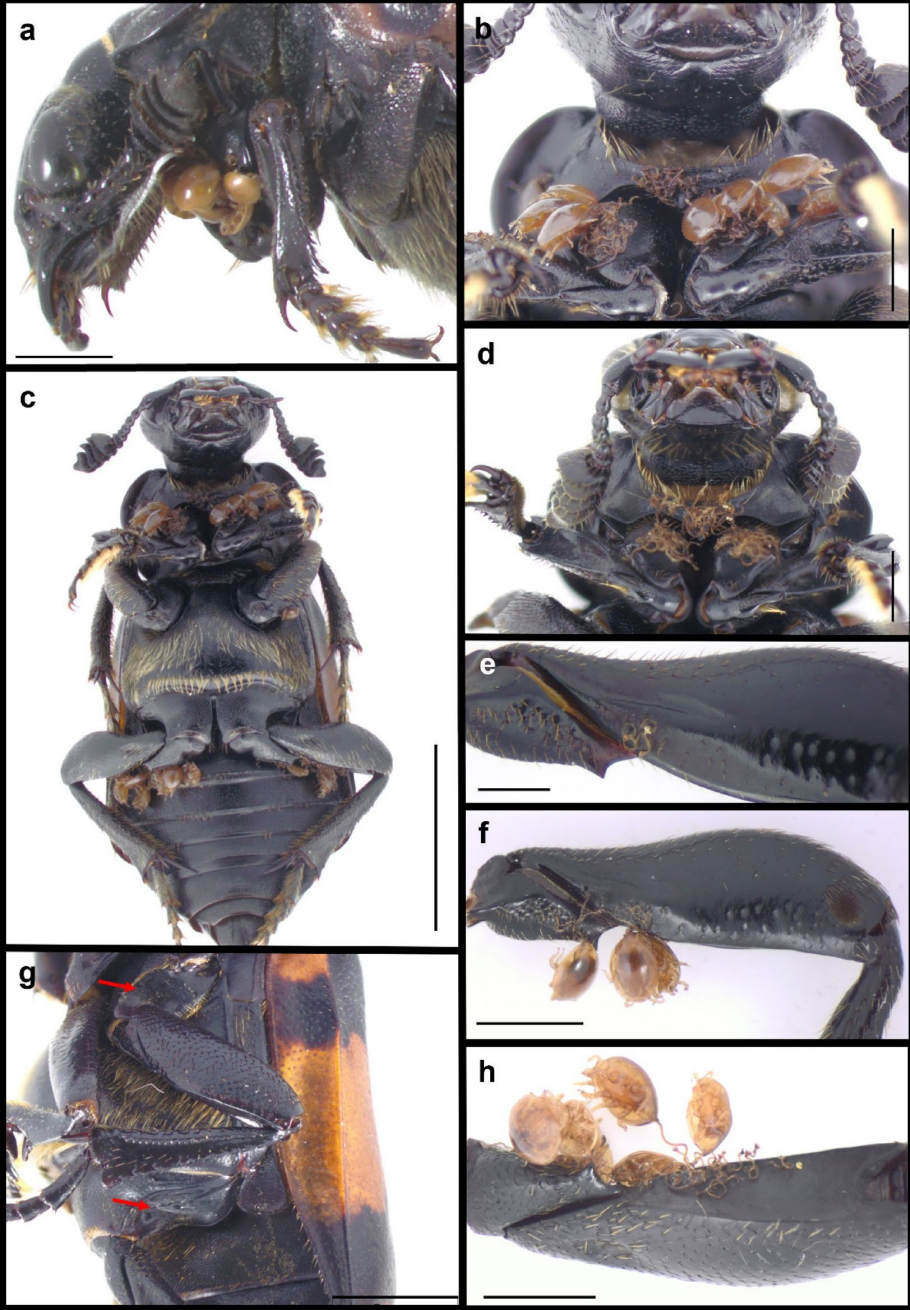
Attachment sites of mites within the *Uroobovella nova* species complex recorded on *Nicrophorus vespilloides*. Deutonymphs attached to the prothorax presternum and ventral sides of the coxae of the forelegs: **a** – Lateral view; **b**, **d** – Ventral view. If the deutonymph is absent, its pedicel remains on the carrier body surface. **c** – Ventral view of *Nicrophorus vespilloides* with attached deutonymphs. **e**, **f**, **h** – Deutonymphs attached to the dorsal side of the femora of the hindlegs, the right side. **g** – Red arrows indicate mesocoxal and metacoxal cavities under which deutonymphs attached to the dorsal sides of the femora are hidden. Scale bars: 2 mm (**a**, **g**); 5 mm (**c**); 1 mm (**b**, **d**, **f**, **h**); 0.5 mm (**e**).

Phoretic deutonymphs were recorded on 56 body parts of *N. vespilloides*, most of which had been infested accidentally (Supplementary Table S2). As many as 45 body sites were infested in less than 10% of the examined beetles, and most frequently, one, two or three deutonymphs were found (Supplementary Table S2). Our analysis revealed a positive correlation between the number of carried deutonymphs and the number of infested beetle body parts (Pearson’s correlation; *r* = 0.72, *n* = 427, *p* < 0.001; Fig. 3). The analysis of the most heavily infested beetle body parts (*n* = 7), considering both the left and right sides of the body as well as the SEX of the beetle, revealed a distinct pattern of mite distribution on the host (GLMM, PART: F = 752.10, df = 6, 2981, *p* < 0.001; Fig. 4), with no significant effect on the beetle’s SEX (GLMM, F = 1.19, df = 1, 2981, *p* = 0.146; Fig. 4). Most deutonymphs were attached to the prothorax presternum, followed by the ventral side of the coxae of the forelegs (Figs. 4 and 5a–d). The next most heavily infested *N. vespilloides* body sites were the dorsal sides of the femora of the hindlegs and midlegs (Figs. 4 and 5c,e,f,h). More rarely, deutonymphs were found on the lateral margins of the proepisternum and dorsal side of the trochanters of the hindlegs (Supplementary Table S2). Deutonymphs were highly specific in the selection of attachment sites (Fig. 5). Within the mid- and hindlegs, mites usually occupied only the area within the lower margin of the dorsal side of the femora, even if the remaining area was not infested (Fig. 5e,f).

## Discussion

Our study revealed that deutonymph infestation in *Nicrophorus vespilloides* was higher than previously observed, indicating highly specific interactions between the studied species^13^. Schwarz et al.^13^ reported that the prevalence of *Uroobovella nova* was 74%, whereas we observed that almost 90% of beetles carried deutonymphs. A striking difference in the deutonymph load is noticeable between our results and those of Schwarz et al.^13^, i.e., five deutonymphs per infested beetle. Interestingly, the high deutonymph prevalence recorded in our study is similar to the previously reported prevalence of *Poecilochirus carabi* (95%) on *N. vespilloides*^13^, and the mite load of *U*. *nova* mites is much higher than that of *P*. *carabi* mites (15.3 deutonymphs per infested beetle)^13^.

Unfortunately, the exact significance of the symbiosis between *U. nova* mites and *N. vespilloides* remains unstudied. According to Athias-Binche et al.^12^, *U*. *nova* mites breed alongside burying beetles, and their life cycles are synchronised. After reaching the brood chamber, deutonymphs detach from parental beetles and moult into adults. The next generation of deutonymphs appears in less than a month and leaves the brood chamber on the parents or juvenile beetles^12^. Deutonymphs rarely moult into adults without beetles and often die after a few weeks. These observations indicate that phoretic dispersal is a critical life strategy in *U*. *nova* mites, which have adapted to live in the brood chamber of their beetle carriers. Thus far, we do not know whether *U*. *nova* derives any other benefits from its relationship with *N. vespilloides* or whether and how this relationship could benefit *N. vespilloides*. According to Neumann and Sellnick^24^, *U. nova* is predatory and probably feeds on eggs and larvae of carrion-colonising flies. If this assumption is accurate, then *U*. *nova*, similarly to *P. carabi* mites, could decrease the number of flies that compete with burying beetles for food resources. Conversely, in the absence of blow flies, *P*. *carabi* mites can also feed on the eggs of burying beetles, reducing their reproductive success^10^. Therefore, future studies should verify the feeding habits of *U*. *nova* mites to determine their exact effects on *N. vespilloides*.

In our study, the deutonymph prevalence was affected only by season, with relatively high proportion of infested beetles occurring in May-July. It is unknown how the temporal dynamics of *N*. *vespilloides* were shaped in our study. Nevertheles, research conducted by Royle & Hopwood^22^ in England and by Kočárek & Benko^25^ in the Czech Republic revealed that, from early May to late July, the abundance of *N*. *vespilloides* is lower than that in August and September. Presumably, the higher deutonymph prevalence in our study in May-July resulted from the lower number of *N*. *vespilloides* individuals at that time. Since the mean number of mites observed on beetles was constant throughout the seasons, more beetles were infested because the mites had fewer carriers to use.

Although the intensity of deutonymph infestation was sex-biased, females had a slightly higher mite load than males did. Since we excluded sexual size dimorphism in *Nicrophorus vespilloides*, this slight difference in deutonymph loads between the sexes may be due to behavioural differences in parental beetles. Compared with males, females of *N. vespilloides* typically spend more time in brood chambers providing food and feeding on larvae^21^, which may increase their exposure to coexisting mites. The effect of prolonged parental care in females on their increased infestation with mites was also reported by De Gasperin et al.^7^ for *N. vespilloides* and *Poecilochirus carabi*. Nevertheless, our findings indicate that selection between carrier sexes is weak (on average 8.8%) in *Uroobovella nova*. If these mites develop only in burying beetle brood chambers, successful dispersal of deutonymphs can be achieved in both sexes because females and males cooperate in carrion utilisation and constructing the nest.

Beetle individuals of different body sizes were selected at similar frequencies by phoretic deutonymphs. In turn, the relationship between the mite load and beetle body size was positive and significant but weak. Generally, our observations align with previous findings, which show that phoretic animals or parasites are more successful on larger hosts [e.g.^6,26,27^]. Moreover, the dispersal of mites on large females of burying beetles is more effective because they have been shown to have greater reproductive success^5^. The effect of body size on the intensity of phoretic individual infestation seems particularly crucial when the dispersal stage is juvenile. More mites can fit on larger carriers which increases the probability of finding a partner after reaching the new habitat. However, the relationships between body size and the mite load are not fully understood, and further research is needed.

Our study revealed considerable temporal variation in deutonymph loads, as hypothesised. In both years, the intensities of deutonymph infestation were similar for both sexes but not in females collected in May–July of 2018 and July–September of 2019. At these time points females presented the highest mite load which could be linked to the extended exposure of *N. vespilloides* females to mites compared with that of males, as explained above. Nevertheless, considering the current knowledge, it is difficult to explain the observed temporal differences in patterns of deutonymph infestation intensity between the sexes in *N*. *vespilloides*. The number of phoretic deutonymphs observed on their beetle carriers is affected by multiple factors, including mite population dynamics and breeding resource availability, which were not analysed in this study^15^. Mite load can also be affected by seasonal differences in the reproductive success of *N. vespilloides*^23^ and differences in the provision of parental care by younger and older individuals^28,29^.

According to Schwarz et al.^13^, the ventral surfaces of the head and forelegs of *Nicrophorus vespilloides* are preferred by deutonymphs of *Uroobovella nova*. This observation partially contradicts our findings because we observed that most deutonymphs were attached to the prothorax presternum and not to the ventral surface of the beetle head. However, the prothorax presternum is located on the ventral side of the body, below the head; therefore, the authors could not precisely determine the exact location of the mites. Deutonymphs of *U. nova* were attached only to the ventral side of the body of *N. vespilloides*. This arrangement protects mites from detachment while they dig into the ground, which has also been observed in other tunneling beetles carrying Uropodina mites, e.g., bark beetles and some dung beetles^30–32^. In addition, burying beetles lie on their dorsal side when moving the carrion, which can cause mites to detach or even die.

It seems that the prothorax presternum, the ventral parts of the coxae, and the dorsal sides of the femora of the hind legs and midlegs were the preferred sites because the deutonymphs were sheltered within them. Deutonymphs attached to the presternum and ventral sides of the the coxae were hidden under the head of the beetle. Deutonymphs attached to the hind and middle legs were partially located within the mesocoxal and metacoxal cavities. The number of deutonymphs attached to *N. vespilloides* decreased towards its posterior body. Interestingly, the hindlegs were more heavily infested than the midlegs. Presumably, owing to its size, the metacoxal cavity provides a much safer area for attachment, explaining the difference in the deutonymph intensity of infestation between the second and third pairs of legs (Fig. 5g). Less frequently, deutonymphs select other body parts, e.g., the left and right lateral margins of the proepisternum, dorsal sides of the trochanters of the hindlegs and posterior parts of the femora of the hindlegs (see Supplementary Table S2).

As we hypothesised, the number of infested beetle body sites was correlated with the number of dispersing deutonymphs. It seems that deutonymphs appeared in many locations only when preferred sites had already been occupied. Presumably, the increasing deutonymph load within sheltered sites also forced subsequent individuals to find similar oppositely located sites. This hypothesis could explain the even distribution of deutonymphs on the right and left sides of the beetle’s body. According to previous observations, deutonymphs of Uropodina disperse in groups and prefer sites already infested by deutonymphs^16,33^. In this way, high attachment site specificity in *U. nova* mites results from the selection of sites that reduce the risk of detachment and intraspecific coaggregation.

Our study presents novel findings on the poorly studied associations between *U*. *nova* and *N. vespilloides.* Highly specific phoretic interactions may have serious ecological and evolutionary consequences for both mites and their carriers. Examples of *Poecilochirus carabi* and *U*. *nova* species complexes show that long-term relationships with their carriers may lead to divergence and speciation in sympatric mite populations^14,34^. *Poecilochirus carabi* mites benefit from *N. vespilloides* as a means of transport but can also be its specialised competitors or parasites, shape parental care, increase reproductive success by eating eggs of blow flies (Calliphoridae), and affect larval mass^7,8,10,11^. Future studies should determine whether and how *U*. *nova* mites affect *N. vespilloides*, particularly its parental care and breeding success. Furthermore, a focus on the interactions between *U*. *nova* and *P*. *carabi* is needed to determine whether they compete for food resources and how their reciprocal presence on *N. vespilloides* affects their fitness.

## Materials and methods

### Study material

Beetles were collected in the Niepołomice Forest, a large forest complex near Kraków, Poland (49°59′–50°07′N, 20°13′–20°28′E, area: 110 km^2^), in 2018 and 2019 as bycatch from saproxylic beetle traps (IBL-2). The IBL-2 flight intercept “window” trap consists of a triangular screen and a funnel affixed to a container filled with ethylene glycol as a preservative. This method of collecting *N*. *vespilloides* with attached *Uroobovella* deutonymphs is convenient for studying phoretic interactions because it prevents the movement of mites between beetles within the same trap and thus reflects natural phoretic patterns. After being submerged in ethylene glycol, the beetles and attached deutonymphs die. However, even if the deutonymphs do not die immediately, their mobility is limited because a pedicel connects them to their beetle carrier. One terminus of the pedicel adheres to the deutonymph’s anus, and the other terminus adheres to the carrier body surface. When detaching from its carrier, the mite swings forward and straightens its legs, which makes contact with the carrier’s body surface possible. The deutonymph then runs forward, which causes detachment of the pedicel from the deutonymph’s anus^16^, and the pedicel usually remains on the carrier body. Such behaviour is impossible when the mite is submerged in ethyl glycol or another preservative, e.g. ethyl alcohol. One hundred traps were set in the study area, and several thousand individuals of *Nicrophorus vespilloides* were caught, which allowed for a random selection of specimens for the study. We analysed individuals of *N. vespilloides* collected during four study periods: July 12, 2018 (insects were trapped from May 30), September 18, 2018 (insects were trapped from July 13), July 2, 2019 (insects were trapped from May 18), and September 5, 2019 (insects were trapped from July 3). We randomly selected 60 females and 60 males of *N. vespilloides* collected during each study period. In total, 480 individuals (240 females and 240 males) of *N. vespilloides* were analysed. The collected material is deposited in the authors’ collections.

### Analysis of mite‒carrier relationships

Deutonymphs and pedicels without deutonymphs found on particular beetle body parts were noted, and their numbers were combined. Many deutonymphs or pedicels without deutonymphs are often attached to one next to the other, forming a tangle (Fig. 5). In such cases, they were first torn off and placed on a paper towel moistened with water and then untangled with an entomological needle so that they could be counted. Deutonymphs were identified via the key for the identification of Karg^35^. The genitalia of all *N. vespilloides* individuals were dissected to ensure accurate determination of sex^36^. The body size of *N. vespilloides* was determined by measuring pronotum width as the distance between its two widest points^37^. Measurements were taken from digital images expressed in mm. Images were taken with an Olympus SZ61 stereomicroscope fitted with a camera and Cell A software (Olympus Corporation, Japan). Males did not differ in size from females (Student’s t-test, t = 0.17, df = 468, *p* = 0.870; males: mean = 4.92, 95% CL: 4.86–4.98, *n* = 234; females: mean = 4.92, 95% CL: 4.86–4.97, *n* = 236).

### Data analyses

We analysed the influence of several factors that likely affect the prevalence (the proportion of infested beetles expressed as a percentage) of deutonymphs on beetles, such as beetles’ SEX, SIZE, SEASON (May–July and July–September) and YEAR (2018 and 2019), as well as the interaction effect of SEX*SEASON and SEX*SEASON*YEAR via a Generalized linear model (GLM) with a binomial distribution and logit link function, where the prevalence (binary variable) was the dependent variable and SEX (binary variable), SIZE (continuous variable), SEASON (categorical variable) and YEAR (categorical variable) were the factors. Furthermore, we analysed the impacts of beetles’ SEX, SIZE, SEASON and YEAR as well as the interaction effects of SEX*SEASON and SEX*SEASON*YEAR on the intensity of deutonymph infestation, e.g., the mean number of deutonymphs per infested beetle, via a GLM with a Poisson distribution and a log link function, where the intensity of mite infestation was the dependent variable and SEX, SIZE, SEASON, YEAR, SEX*SEASON and SEX*SEASON*YEAR were the factors. Finally, we examined the distribution of mites on beetles, considering the most infested parts of their bodies [PART], taking into account their left or right side (*n* = 7) and their SEX via a Generalized linear mixed model (GLMM) with a Poisson distribution and log link function, where the number of mites was the dependent variable, PART and SEX were fixed factors, and the beetle ID was a random factor. All the calculations were performed via IBM SPSS Statistics for Windows^38^. Throughout the text, the mean values are presented with 95% confidence limits (CL).

## Electronic supplementary material

Below is the link to the electronic supplementary material.

Supplementary Material 1

## Data Availability

The data that support the findings of this study are available from the corresponding authors upon reasonable request.
