# Supplementary material for: Influences of carrier sex, body size, and time on the symbiotic interaction between Nicrophorus vespilloides and the Uroobovella nova mite species complex
Source: Sci Rep. 2025 Jun 5;15:19823. doi: 10.1038/s41598-025-04685-y (PMC12141562; doi:10.1038/s41598-025-04685-y)
Supplement: Supplementary file 1 — Supplementary Material 1 [file 41598_2025_4685_MOESM1_ESM.pdf]

Influences of carrier sex, body size, and time on the symbiotic interaction between *Nicrophorus vespilloides* and the *Uroobovella nova* mite species complex

Daria Bajerlein, Piotr Zduniak, Aleksandra Wyszyńska, Edward Baraniak, Marek Przewoźny, Tomasz Grzegorczyk, Arkadiusz Urbański

Supplementary Table S1. Prevalence (P) of deutonymphs within the *Uroobovella nova* species complex phoretic on *Nicrophorus vespilloides*.

Nicrophorus vespilloides	**n**	**P (%)**	**95% CL**
Males	215	90.3	85.85 – 93.77
Females	212	88.3	83.58 – 92.11
Individuals collected in May–July	221	92.5	88.36 – 95.48
Individuals collected in July–September	206	86.2	81.16 – 90.30
Individuals collected in 2018	217	90.8	86.40 – 94.14
Individuals collected in 2019	210	87.9	83.04 – 91.72

n – the number of infested individuals of beetles; CL – confidence limits calculated from the binomial distribution.

Supplementary Table S2. Body sites of *Nicrophorus vespilloides* infested with deutonymphs of the *Uroobovella nova* species complex.

Beetle body sites	n	P (%)	95% CL	mean ± SD	range
Prothorax presternum	413	86.4	83.00 – 89.35	6.3 ± 4.32	1 – 23
The ventral part of the coxa, left foreleg	398	83.3	79.61 – 86.50	5.7 ± 4.36	1 – 24
The ventral part of the coxa, right foreleg	388	81.2	77.37 – 84.58	6.0 ± 4.90	1 – 25
The dorsal part of the femur, left hindleg	209	43.7	39.22 – 48.30	2.5 ± 2.00	1 – 13
The dorsal part of the femur, right hindleg	198	41.4	36.97 – 45.99	2.7 ± 2.33	1 – 15
The dorsal part of the femur, left midleg	127	26.6	22.66 – 30.77	2.4 ± 2.35	1 – 14
The dorsal part of the femur, right midleg	93	19.5	16.00 – 23.29	2.3 ± 2.01	1 – 13
The lateral margin of proepisternum, left side	80	16.7	13.50 – 20.39	2.5 ± 2.11	1 – 11
The lateral margin of proepisternum, right side	69	14.4	11.41 – 17.91	2.6 ± 2.18	1 – 13
The dorsal part of the trochanter, right hindleg	69	14.4	11.41 – 17.91	1.9 ± 1.41	1 – 8
The dorsal part of the trochanter, left hindleg	65	13.6	10.65 – 17.00	2.1 ± 2.16	1 – 15
The posterior part of the femur, right hindleg	41	8.6	6.23 – 11.46	1.4 ± 0.89	1 – 6
The posterior part of the femur, left hindleg	38	7.9	5.69 – 10.75	1.2 ± 0.75	1 – 5
The ventral part of the femur, left foreleg	26	5.4	3.58 – 7.87	1.8 ± 1.65	1 – 7
The ventral part of the femur, right foreleg	23	4.8	3.07 – 7.13	1.5 ± 1.12	1 – 6
The posterior part of the femur, left midleg	21	4.4	2.74 – 6.64	1.2 ± 0.54	1 – 3
The posterior part of the femur, right midleg	15	3.1	1.77 – 5.12	1.1 ± 0.35	1 – 2
The posterior part of the femur, left foreleg	11	2.3	1.15 – 4.08	1.2 ± 0.60	1 – 3
The posterior part of the femur, right foreleg	9	1.9	0.86 – 3.54	1.3 ± 0.50	1 – 2
1–st abdominal sternite, left side	7	1.5	0.59 – 2.99	1.1 ± 0.38	1 – 2
1–st abdominal sternite, right side	7	1.5	0.59 – 2.99	1.3 ± 0.76	1 – 3
The dorsal part of the femur, left foreleg	6	1.3	0.46 – 2.71	3.0 ± 2.76	1 – 7
The dorsal part of the femur, right foreleg	6	1.3	0.46 – 2.71	4.0 ± 4.69	1 – 13
Prosternum, right side	6	1.3	0.46 – 2.71	2.0 ± 1.26	1 – 4
Right metepimeron	5	1.0	0.34 – 2.42	1.0 ± 0.00	1 – 1
The ventral part of the femur, left midleg	5	1.0	0.34 – 2.42	1.6 ± 0.89	1 – 3
Prosternum, left side	5	1.0	0.34 – 2.42	1.8 ± 1.30	1 – 4
Right proepimeron	5	1.0	0.34 – 2.42	1.6 ± 0.89	1 – 3
The ventral part of the femur, right midleg	4	0.8	0.23 – 2.13	3.0 ± 4.00	1 – 9
Left proepimeron	4	0.8	0.23 – 2.13	1.5 ± 0.58	1 – 2
The dorsal part of the coxa, left foreleg	3	0.6	0.13 – 1.82	1.0 ± 0.00	1 – 1
The ventral part of the femur, left hindleg	3	0.6	0.13 – 1.82	1.0 ± 0.00	1 – 1
The ventral part of the coxa, right hindleg	3	0.6	0.13 – 1.82	1.7 ± 1.15	1 – 3
The ventral part of the femur, right hindleg	3	0.6	0.13 – 1.82	1.0 ± 0.00	1 – 1
The ventral part of the trochanter, right hindleg	3	0.6	0.13 – 1.82	1.0 ± 0.00	1 – 1
2-nd abdominal sternite, left side	2	0.4	0.05 – 1.50	1.0 ± 0.00	1 – 1
Left metepimeron	2	0.4	0.05 – 1.50	1.0 ± 0.00	1 – 1
The dorsal part of the trochanter, right midleg	2	0.4	0.05 – 1.50	1.0 ± 0.00	1 – 1

The ventral part of the coxa, left hindleg	2	0.4	0.05 – 1.50	1.0 ± 0.00	1 – 1
The dorsal part of the coxa, left midleg	2	0.4	0.05 – 1.50	1.0 ± 0.00	1 – 1
The right side of the mesosternum	2	0.4	0.05 – 1.50	1.0 ± 0.00	1 – 1
3-rd abdominal sternite, left side	1	0.2	0.01 – 1.16	1.0 ± 0.00	1 – 1
3-rd abdominal sternite, right side	1	0.2	0.01 – 1.16	1.0 ± 0.00	1 – 1
The left side of the metasterum	1	0.2	0.01 – 1.16	1.0 ± 0.00	1 – 1
The right side of the metasternum	1	0.2	0.01 – 1.16	1.0 ± 0.00	1 – 1
The dorsal part of the coxa, right hindleg	1	0.2	0.01 – 1.16	1.0 ± 0.00	1 – 1
The dorsal part of the trochanter, right foreleg	1	0.2	0.01 – 1.16	1.0 ± 0.00	1 – 1
The ventral part of the coxa, left midleg	1	0.2	0.01 – 1.16	1.0 ± 0.00	1 – 1
The ventral part of the coxa, right midleg	1	0.2	0.01 – 1.16	1.0 ± 0.00	1 – 1
The dorsal part of the trochanter, left midleg	1	0.2	0.01 – 1.16	1.0 ± 0.00	1 – 1
The ventral part of the trochanter, left hindleg	1	0.2	0.01 – 1.16	1.0 ± 0.00	1 – 1
The middle part of the metasterum	1	0.2	0.01 – 1.16	1.0 ± 0.00	1 – 1
2-nd abdominal sternite, right side	1	0.2	0.01 – 1.16	1.0 ± 0.00	1 – 1
The dorsal part of the pronotum	1	0.2	0.01 – 1.16	1.0 ± 0.00	1 – 1
Tibia, right midleg	1	0.2	0.01 – 1.16	1.0 ± 0.00	1 – 1
1-st abdominal sternite, middle part	1	0.2	0.01 – 1.16	1.0 ± 0.00	1 – 1

n – the number of beetle individuals with deutonymphs attached to a given body site; P – deutonymph prevalence expressed as a percentage of beetles with deutonymphs attached to a given body site; CL– confidence intervals calculated from the binomial distribution; the mean number of deutonymphs attached to a given body site; SD – standard deviation.
